# Supplementary material for: AIP limits neurotransmitter release by inhibiting calcium bursts from the ryanodine receptor
Source: Nat Commun. 2017 Nov 9;8:1380. doi: 10.1038/s41467-017-01704-z (PMC5680226; doi:10.1038/s41467-017-01704-z)
Supplement: Supplementary file 1 — Supplementary Information [file 41467_2017_1704_MOESM1_ESM.pdf]

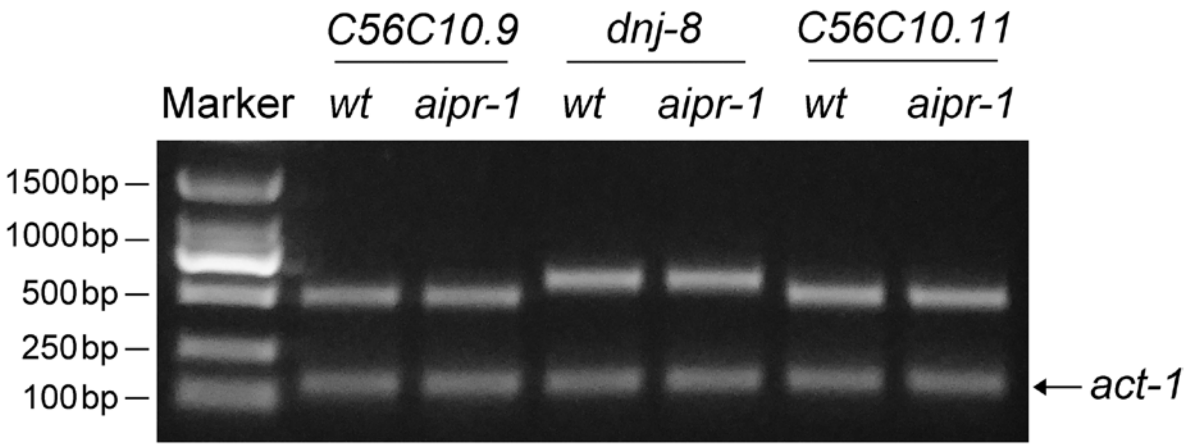

Supplementary Figure 1. ***aipr-1(zw86)*** **mutation did not alter transcript levels of the other three genes in the operon.** Total RNA was extracted using Trizol reagent. RT-PCR was performed using gene-specific primers. RT-PCR was also performed for *act-1* to indicate similar amount of cDNA template. C56C10.11 is likely to be part of the *epg-5* gene based on gene conservation.

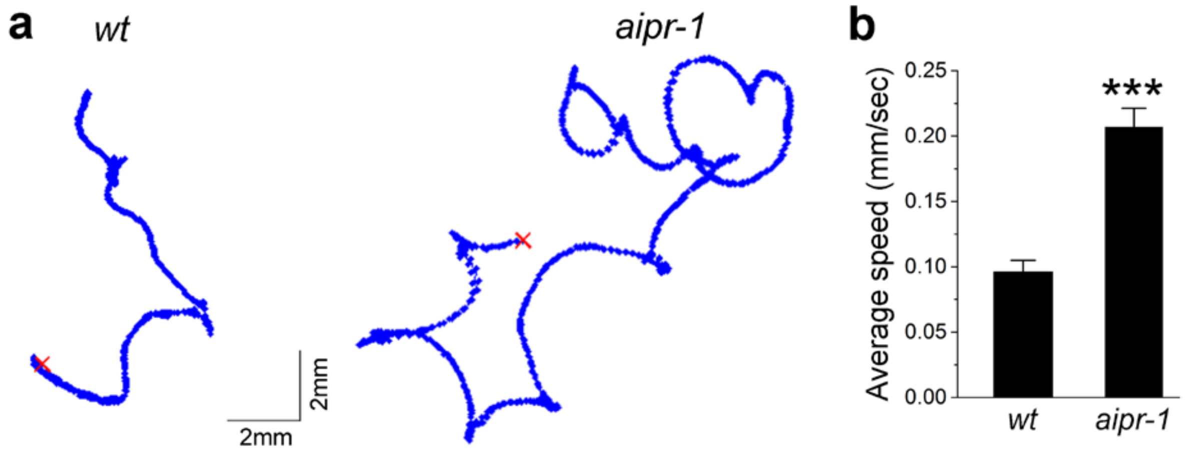

Supplementary Figure 2. ***aipr-1(zw86)*** **mutant worms move faster than the wild type (*wt*). a,** Locomotion path of representative worms over 10 minutes on agar plate seeded with a thin layer of OP50. The locomotion track was reconstructed by *Track-A-Worm*, an automated worm tracker used for the locomotion analyses. In each assay, a single young adult worm was placed in the center of an OP50 bacterial lawn, and locomotion behavior was recorded for 10 minutes after a 15-minute recovery time from the transfer. The red “x” marks the starting point. **b,** Comparison of locomotion speed between *wt* (n = 11) and *aipr-1(zw86)* mutant (n = 11). Data are shown as mean ± s.e.m. *** $p < 0.001$ compared with *wt* (unpaired *t*-test).

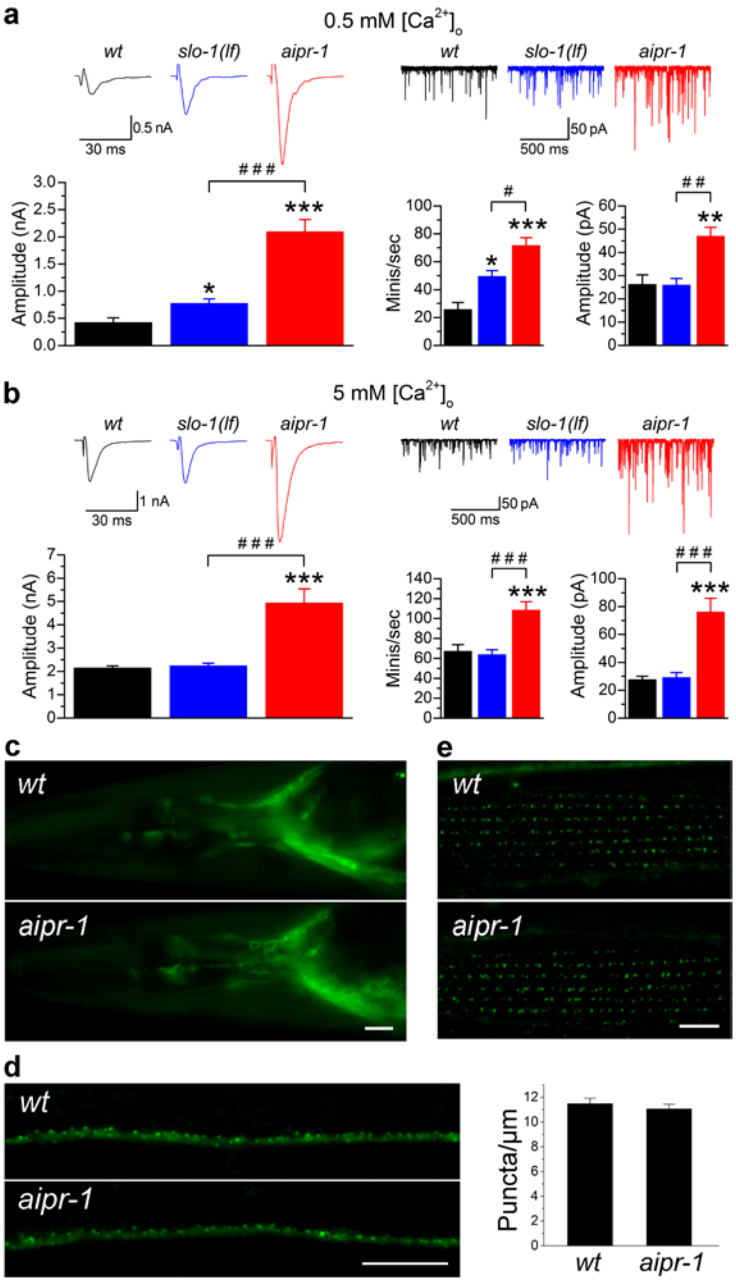

Supplementary Figure 3. AIPR-1 does not act through SLO-1 in regulating presynaptic release at the neuromuscular junction. a, Comparison of evoked currents and spontaneous miniature currents (minis) recorded at 0.5 mM $[Ca^{2+}]_o$ (Extracellular solution II) among wild type (*wt*), *slo-1(md174)* (putative null), and *aipr-1(zw86)*. n = 8 in all groups. **b,** Comparison of evoked currents and spontaneous minis recorded at 5 mM $[Ca^{2+}]_o$ (Extracellular solution I) among the three groups (*wt*, n = 8; *slo-1(md1745)*, n = 9; *aipr-1(zw86)*, n = 7). **c-e,** Deficiency of *aipr-1* does not alter SLO-1 expression or subcellular localization. SLO-1::GFP, which was expressed under the control of P*slo-1*, is indistinguishable between *wt* and *aipr-1(zw86)*. Shown are representative images of the head region (**c**), a segment of the dorsal nerve cord (**d**), and body-wall muscle (**e**). The density of SLO-1::GFP puncta in **d** was compared between *wt* (n = 21) and *aipr-1(zw86)* (n = 20). Data are shown as mean ± s.e.m. **P* < 0.05, ***P* < 0.01, ****P* < 0.001 compared with *wt*; #*P* < 0.05, ##*P* < 0.01, ###*P* < 0.001 compared between *slo-1(lf)* and *aipr-1(zw86)* (one-way ANOVA followed by Tukey's post hoc test). Scale bars, 10 μm.

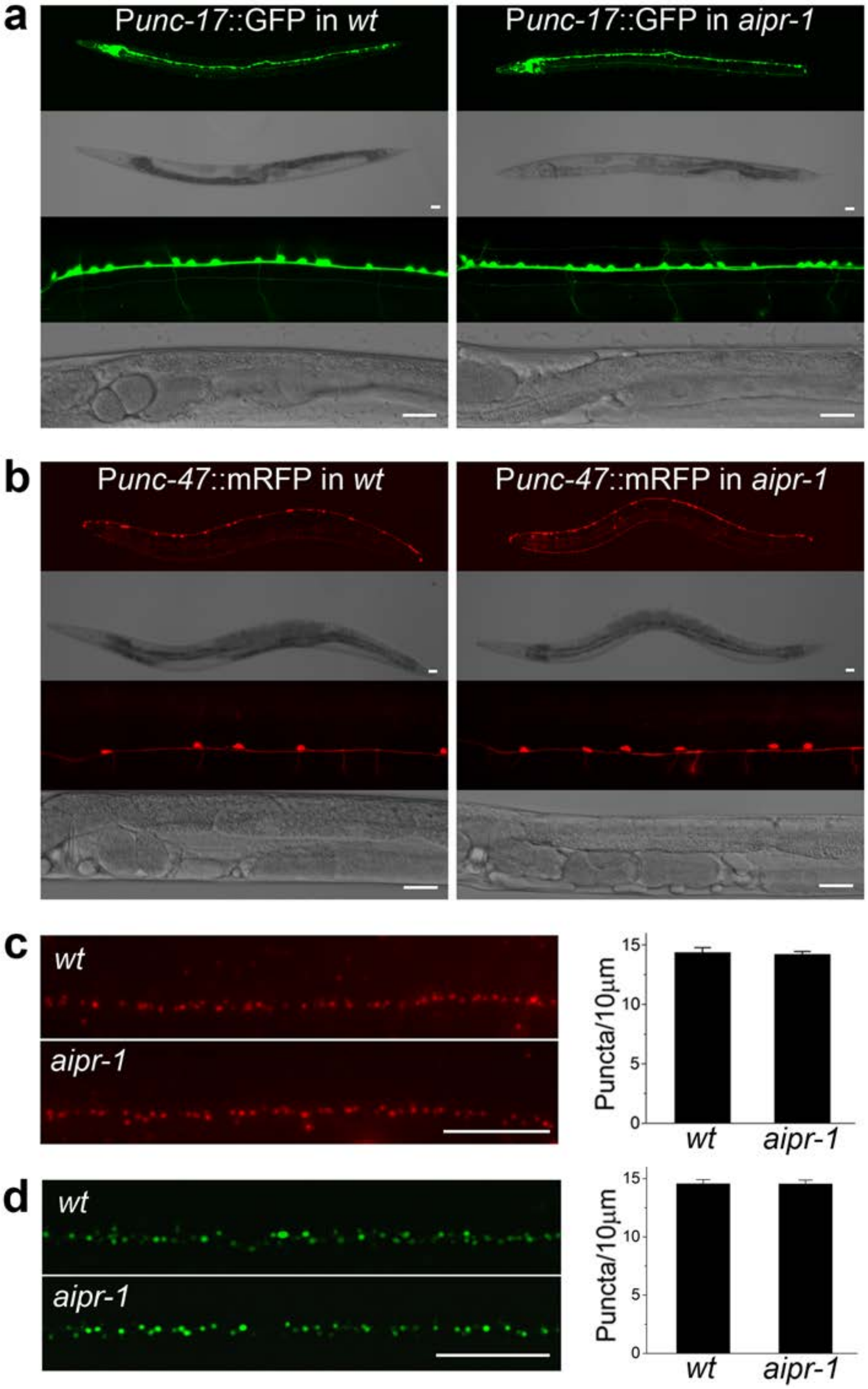

Supplementary Figure 4. Motor neuron gross morphology and synapse density are similar between *aipr-1(zw86)* and wild type (*wt*). a, b, Representative images of GFP-labeled acetylcholine motor neurons (**a**) and mRFP-labeled GABA motor neurons (**b**), and corresponding worm DIC images of *wt* and *aipr-1(zw86)*. GFP and mRFP were expressed under the control of P*unc-17* and P*unc-47*, respectively. **c** and **d**, Representative images of RIM-immunoreactive puncta (**c**, *wt* n = 22; *aipr-1* n = 27) and GFP::ELKS-1 puncta (**d**, *wt* n = 27; *aipr-1* n = 24) in the dorsal nerve cord of *wt* and *aipr-1(zw86)*, and statistical comparison of punctum density between the two groups. Scale bars, 20 µm (**a, b**); 10 µm (**c, d**).

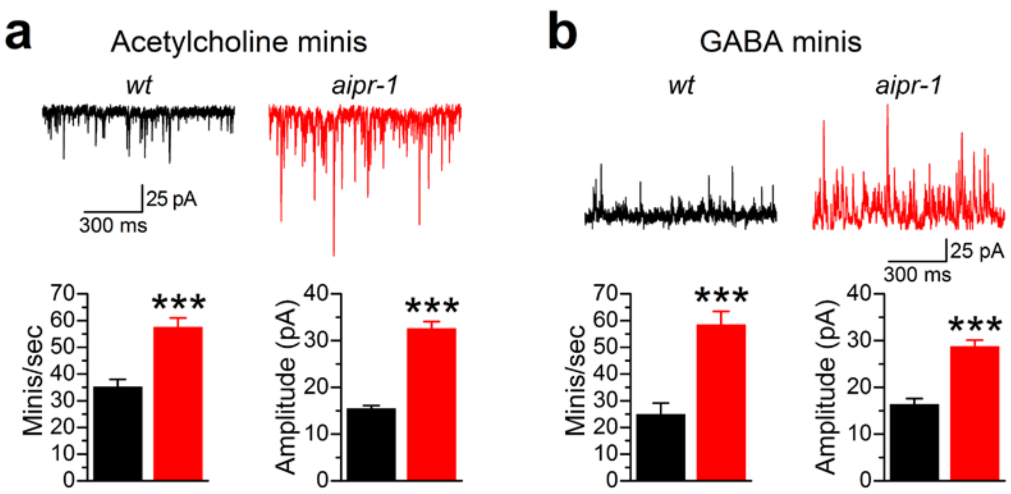

Supplementary Figure 5. AIPR-1 deficiency augmented both acetylcholine and GABA spontaneous minis. a, Comparison of acetylcholine spontaneous miniature currents (minis) between wild type (*wt*) (n = 9) and *aipr-1(zw86)* (n = 9). **b,** Comparison of GABA minis between *wt* (n = 8) and *aipr-1(zw86)* (n = 9). Extracellular solution I and pipette solution II were used in these experiments with the membrane voltage held at -60 mV and -10 mV to record acetylcholine and GABA (upward deflections) minis, respectively. Data are shown as mean ± s.e.m. *** $p < 0.001$ compared with *wt* (unpaired *t*-test).

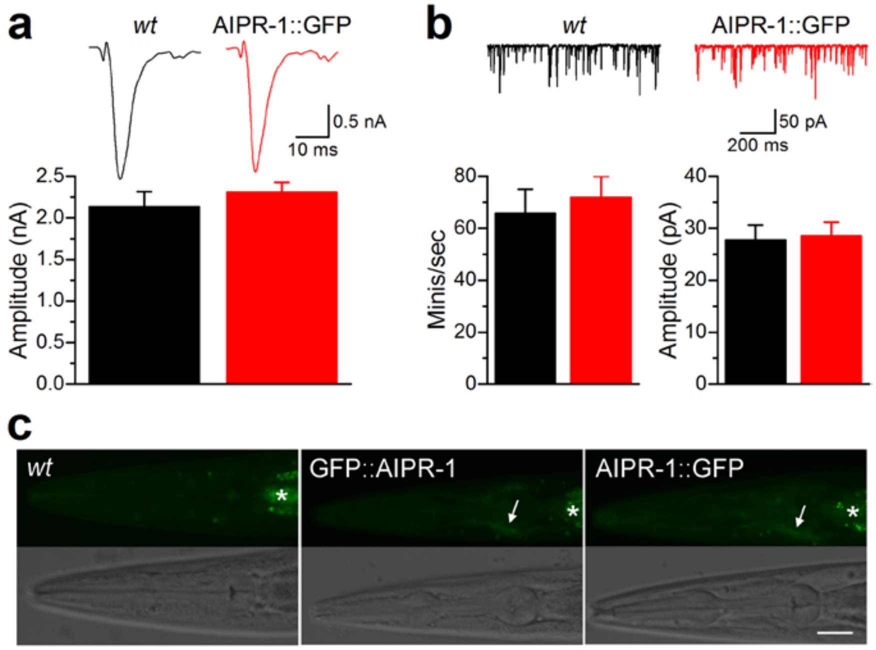

Supplementary Figure 6. Endogenous AIPR-1 tagged by GFP using the CRISPR/Cas9 approach is functional. a and **b,** Evoked responses (**a**) and spontaneous miniature currents (minis) (**b**) at the neuromuscular junction were similar between wild type (*wt)* (n = 8) and the strain with AIPR-1 tagged at the carboxyl terminus (AIPR-1::GFP) (n = 8). **c,** Weak GFP signal was observed in the head region but not elsewhere in both the AIPR-1::GFP strain and a strain with AIPR-1 tagged at the amino terminus (GFP::AIPR-1). No GFP signal was detected in *wt*. The asterisk (*) marks autofluorescence from the gut. Scale bar, 20 μm.

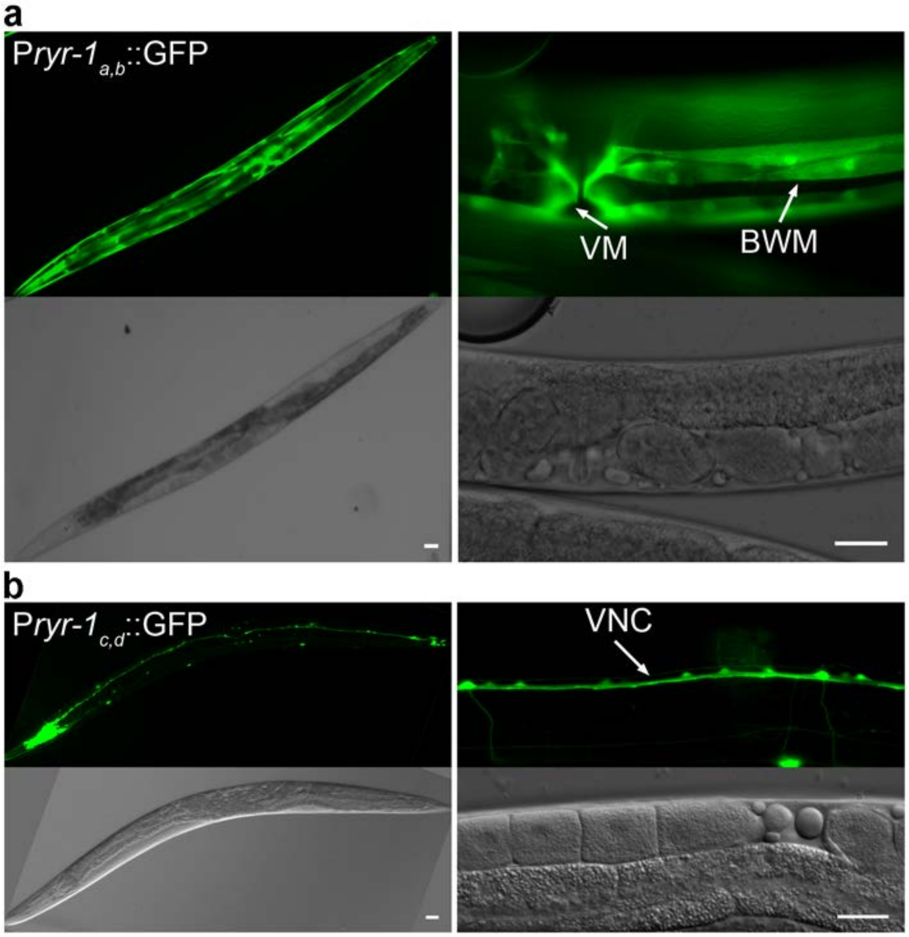

Supplementary Figure 7. RYR-1 is expressed in muscles and neurons. The *ryr-1* gene encodes four alternatively spliced isoforms (*a, b, c, d*) with two different initiation sites (www.wormbase.org). **a,** A GFP reporter construct containing 2.5 kb sequence upstream of the initiation site of *ryr-1a* and *ryr-1b* (P*ryr-1*$_{a,b}$::GFP) drives expression in body-wall muscles (BWM) and vulva muscles (VM). **b,** A GFP reporter construct containing 4.8 kb sequence upstream of the initiation site of *ryr-1c* and *ryr-1d* (P*ryr-1*$_{c,d}$::GFP) drives expression in many neurons, including motor neurons in the ventral nerve cord (VNC). Scale bars, 20 µm.

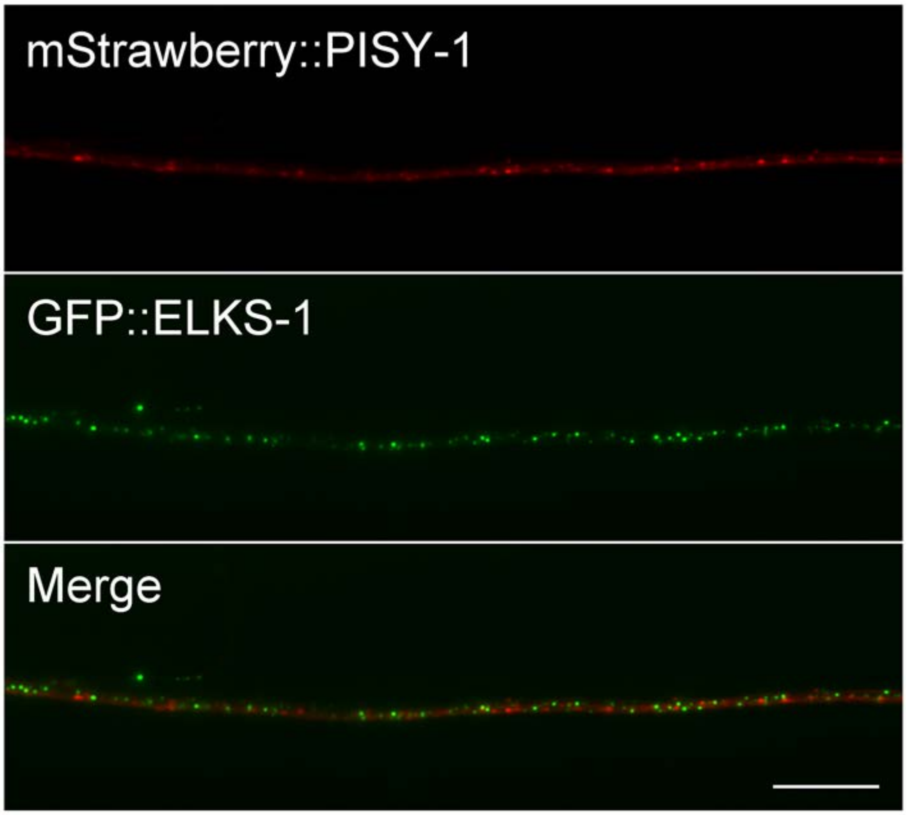

Supplementary Figure 8. The endoplasmic reticulum (ER) extends throughout the dorsal nerve cord and is close to presynaptic sites. The ER was labeled by mStrawberry::PISY-1 while presynaptic sites by GFP::ELKS-1. mStrawberry signal was diffuse with some enriched puncta, which probably result from aggregates of the fusion protein, whereas GFP signal mainly appeared as puncta. The merged picture shows that GFP-labeled presynaptic sites overlap with mStrawberry-labeled ER. Scale bar, 10 µm.

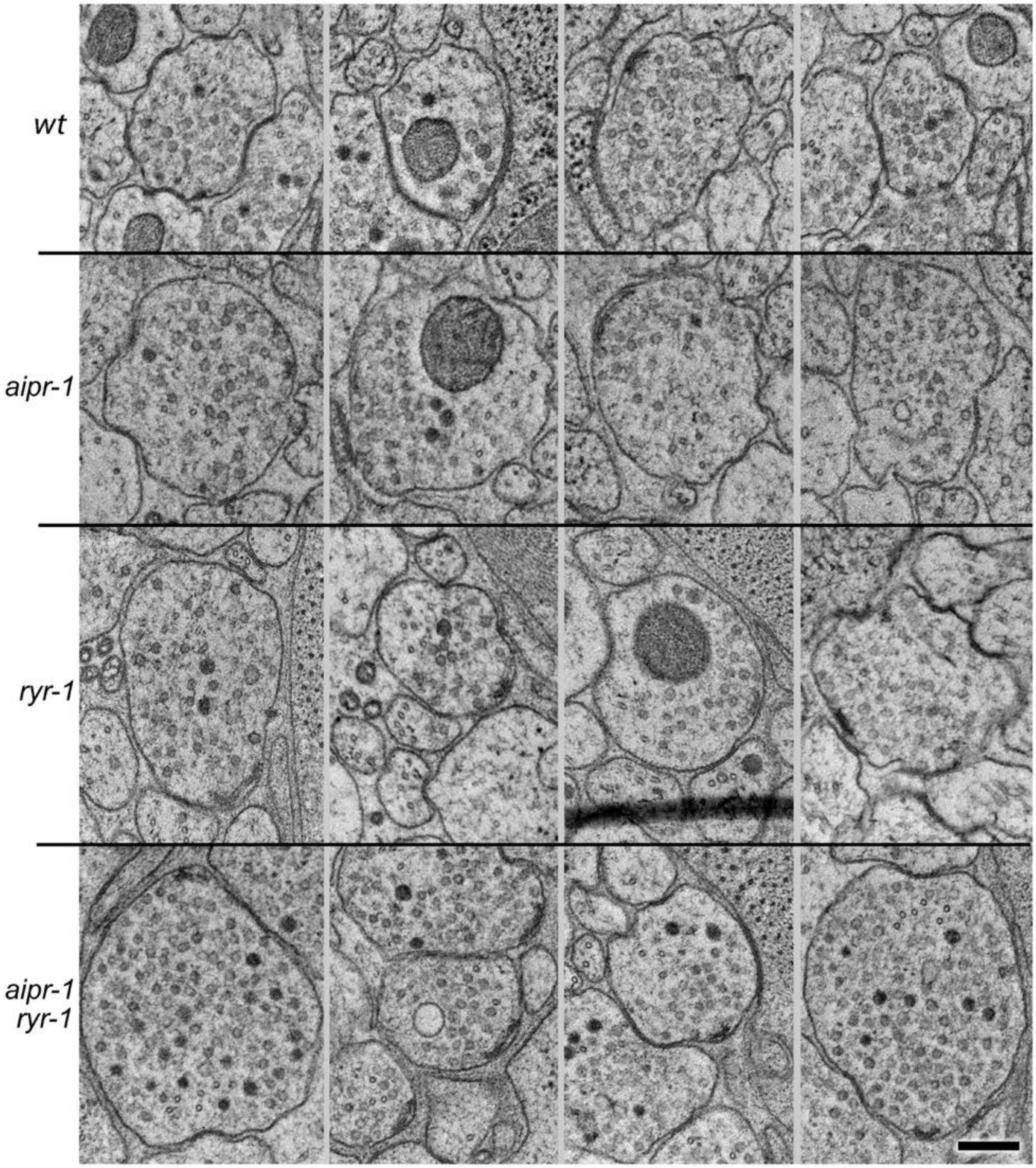

Supplementary Figure 9. Electron micrographs. Sample images from acetylcholine synapses of the wild type (*wt*), *aipr-1(zw86)*, *ryr-1(e540),* and *aipr-1(zw86) ryr-1(e540)* double mutants. Scale bar 200 nm.

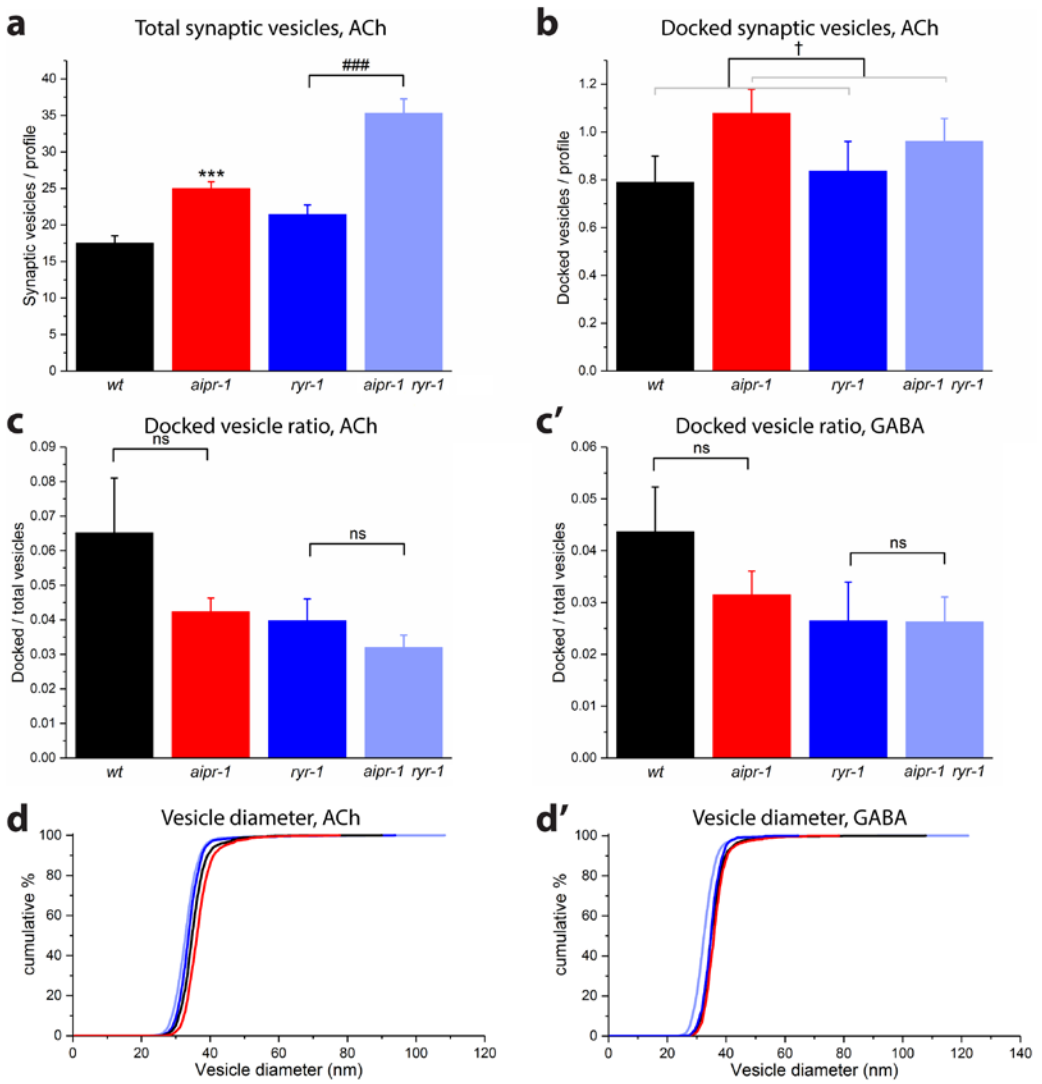

Supplementary Figure 10. *aipr-1(zw86)* acetylcholine synapses display the same phenotypes as GABA synapses. a and **b,** At acetylcholine synapses, *aipr-1(zw86)* increases synaptic vesicle numbers (a) and docked synaptic vesicles (**b**) regardless of the *ryr-1* genotype (total vesicles: *wt* 17.5 ± 1.0 ; *aipr-1* 25.0 ± 0.9; *ryr-1* 21.4 ± 1.3; *aipr-1 ryr-1* 35.2 ± 1.9; docked vesicles: *wt* 0.8 ± 0.1; *aipr-1* 1.1 ± 0.1; *ryr-1* 0.8 ± 0.1; *aipr-1 ryr-1* 1.0 ± 0.1). The increase in synaptic vesicles is profound in the *aipr-1 ryr-1* double mutant in acetylcholine neurons; it is not clear whether this is due to the small data set possible by EM, to a synthetic defect of the mutations, or to a specific response of the acetylcholine synapses. **c**, *aipr-1(zw86)* does not significantly affect the ratio of docked/total synaptic vesicles in both acetylcholine (**c**) and GABA (**c'**) synapses (ACh: *wt* 0.065 ± 0.016 ; *aipr-1* 0.042 ± 0.004; *ryr-1* 0.040 ± 0.006; *aipr-1 ryr-1* 0.032 ± 0.004; GABA: *wt* 0.044 ± 0.009; *aipr-1* 0.032 ± 0.005; *ryr-1* 0.026 ± 0.007; *aipr-1 ryr-1* 0.026 ± 0.005). **d**, Cumulative distribution plots of synaptic vesicle diameters in acetylcholine (**d**) and GABA (**d'**) neurons (mean diameter ACh: *wt* 34.6 ± 0.8 nm ; *aipr-1* 36.9 ± 0.8 nm; *ryr-1* 34.0 ± 0.7 nm; *aipr-1 ryr-1* 32.4 ± 0.5 nm; GABA: *wt* 35.3 ± 0.8 nm; *aipr-1* 36.0 ± 0.6 nm; *ryr-1* 34.4 ± 0.7 nm; *aipr-1 ryr-1* 33.1 ± 0.6). Acetylcholine synapses compared among *wt* (n = 76 synaptic profiles), *aipr-1(zw86)* (n = 102), *ryr-1(e540)* (n = 55), and *aipr-1(zw86) ryr-1(e540)* (n = 101). Data are shown as mean ± s.e.m. *** $p < 0.001$ compared with *wt*; $^{\#\#\#}p < 0.001$, ns $p > 0.05$ compared between indicated groups. (**a,c,d**) Welch's two-tailed *t*-test, $^{\dagger}p < 0.05$ *aipr-1(zw86)* effect via generalized linear model, Poisson family (*ryr-1(e540)* effect: ns) (**d**) *aipr-1* compared with *wt* (ACh) $p = 0.04$, all other comparisons ns.
