# Supplementary material for: AIP limits neurotransmitter release by inhibiting calcium bursts from the ryanodine receptor
Source: Nat Commun. 2017 Nov 9;8:1380. doi: 10.1038/s41467-017-01704-z (PMC5680226; doi:10.1038/s41467-017-01704-z)
Supplement: Supplementary file 3 — Description of Additional Supplementary Files [file 41467_2017_1704_MOESM3_ESM.pdf]

File Name: Supplementary Movie 1
Description: Thrashing of *wt*

File Name: Supplementary Movie 2
Description: Thrashing of *aipr-1(zw86)*

File Name: Supplementary Movie 3
Description: Thrashing of *ryr-1(e540)*

File Name: Supplementary Movie 4
Description: Thrashing of *aipr-1(zw86) ryr-1(e540)*

File Name: Supplementary Movie 5
Description: Thrashing of *unc-2(e55)*

File Name: Supplementary Movie 6
Description: Thrashing of *aipr-1(zw86) unc-2(e55)*

File Name: Supplementary Movie 7
Description: Calcium transients of wild type

File Name: Supplementary Movie 8
Description: Calcium transients of *aipr-1* RNAi

File Name: Supplementary Movie 9
Description: Calcium transients of *ryr-1* RNAi

File Name: Supplementary Movie 10
Description: Calcium transients of *aipr-1 ryr-1* double RNAi
